# Post-translational regulation of the cleaved fragment of Par-4 in ovarian and endometrial cancer cells

**DOI:** 10.18632/oncotarget.9235

**Published:** 2016-05-09

**Authors:** Kevin Brasseur, François Fabi, Pascal Adam, Sophie Parent, Laurent Lessard, Eric Asselin

**Affiliations:** ^1^ Research Group in Cellular Signaling, Department of Medical Biology, Université du Québec à Trois-Rivières, Trois-Rivières, Québec G9A 5H7, Canada

**Keywords:** Par-4, PI3K, MAPK, proteasome, gynaecological cancers

## Abstract

We recently reported the caspase3-dependent cleavage of Par-4 resulting in the accumulation of a 25kDa cleaved-Par-4 (cl-Par-4) fragment and we investigated in the present study the mechanisms regulating this fragment using cl-Par-4-expressing stable clones derived from ovarian and endometrial cancer cell lines.

Cl-Par-4 protein was weakly express in all stable clones despite constitutive expression. However, upon cisplatin treatment, cl-Par-4 levels increased up to 50-fold relative to baseline conditions. Treatment of stable clones with proteasome and translation inhibitors revealed that cisplatin exposure might in fact protect cl-Par-4 from proteasome-dependent degradation. PI3K and MAPK pathways were also implicated as evidenced by an increase of cl-Par-4 in the presence of PI3K inhibitors and a decrease using MAPK inhibitors. Finally using bioinformatics resources, we found diverse datasets showing similar results to those we observed with the proteasome and cl-Par-4 further supporting our data.

These new findings add to the complex mechanisms regulating Par-4 expression and activity, and justify further studies addressing the biological significance of this phenomenon in gynaecological cancer cells.

## INTRODUCTION

In North America and Europe, gynaecological cancer accounts for more than 1/10^th^ of cancer deaths and new cases among women [1–3]. Ovarian cancer is the fifth leading cause of cancer death among women and is the gynaecological cancer causing the highest mortality rate [1–5]. Endometrial cancer is the most common gynaecological cancer with the highest rate of new cases each year [1–3, 6]. One major hurdle among feminine cancers is that advanced and recurrent cases often come with acquired chemoresistance that drastically reduces patient survival rates [5, 6]. New molecular targets are thus required to eliminate recurrence and overcome chemoresistant cancers.

Prostate apoptosis response-4 (Par-4) is one potential therapeutic protein because of its unique ability to induce apoptosis only in cancer cells in a selective manner [7, 8]. Par-4 unique apoptotic ability is activated by numerous complex mechanisms including the intrinsic and extrinsic caspase pathways [8–10]. Based on the human protein atlas, a considerably high level of Par-4 mRNA and protein can be found in both endometrium and ovary tissue relative to other tissue types [11]. It has been shown that Par-4 knock-out mice have a reduced lifespan and more than 36% of the studied animals developed endometrial cancer after only one year of living [12]. Nevertheless, except for a few studies, very little is known about Par-4 in ovarian and endometrial tissues [13–21]; it is also interesting to note that half of the studies were conducted in normal instead of cancer tissues [18–21]. The role and regulation of Par-4 in ovarian and endometrial malignancies thus warrants further investigation.

The functions and subcellular localization of Par-4 is regulated by various mechanisms. First, Par-4 is phosphorylated at Thr163 (Thr155 in rat) by PKA allowing the protein to translocate to the nucleus and induce apoptosis in cancer cells [22]. Localization plays a critical role in Par-4 ability to induce apoptosis. Indeed, Par-4 needs to translocate to the nucleus, via its NLS2 domain, to activate the apoptotic cascade and phosphorylation by PKA at Thr163 is a pre-requisite for this nuclear entry [8]. In the case of normal cells, they express a moderate to high level of Par-4, however most of the protein is located in the cytoplasm [11, 21, 23]. Par-4 also has a second site of phosphorylation at Ser249 in rats which is phosphorylated by AKT1 [24]. AKT1 can also bind and phosphorylates Par-4 in human cells, however, the exact site of phosphorylation in the human sequence of Par-4 has yet to be experimentally confirmed. AKT1 binds directly on Par-4's leucine zipper domain and then phosphorylates Par-4 to maintain the protein in the cytoplasm leading to cancer cells survival and inhibition of Par-4 apoptotic activity [24]. In turn, Par-4 is also known to negatively regulate the level/activity of AKT downstream targets such as NFκB and XIAP [25–27]. Data from cbioportal.org indicates that PI3K/AKT/PTEN pathway is more than often mutated or amplified in endometrial (>90%) and ovarian cancers (>55%), offering an advantage for cancer cell survival [28, 29]. These high levels of alteration in the PI3K/AKT/PTEN pathway indicate a potential issue for Par-4 activity, because of AKT1 negative regulation, in endometrial and ovarian cancers and are also known for being important key protein related to the chemoresistance of the feminine cancers [5, 6, 30–32]. Par-4 downregulation can also increase the components of the PI3K/AKT pathway, conferring resistance to chemotherapy to pancreatic cancer cells [33]. While previous publications have hinted at the crosstalk between the PI3K pathway and Par-4 dynamics, very little mechanistic work has been made toward the clarification of this relationship [24, 34].

In addition to phosphorylation, Par-4 is also regulated by other post-translational mechanisms. Indeed, a recent paper has shown that Par-4 can be ubiquitinated via binding with Fbxo45 protein on its VASA domain to decrease its protein level [35]. We also recently discovered that Par-4 is cleaved by caspase-3 at D131 during apoptosis in many different cancer cells. This cleavage consistently generates a 25kDa cleaved fragment (cl-Par-4) that contains the nuclear localization sequence (NLS2), the selective for apoptosis induction in cancer cells domain (SAC) and the leucine zipper domains [14]. Most importantly, the accumulation of cl-Par-4 under various apoptotic stimuli seems to be an important factor related to the chemosensitivity of cancer cells and the level of apoptosis observed. Nevertheless, beside a few incidental reports in the literature [36–38], not much is known about this cleaved fragment. Considering the potential importance of this modification, further exploration of the mechanisms related to this fragment are required to better understand Par-4 functions. In this study, we report for the first time that cl-Par-4 is regulated by diverse post-translational mechanisms including the proteasome and the PI3K/MAPK survival pathways in both ovarian and endometrial cancer.

## RESULTS

### Par-4 is cleaved only in chemosensitive ovarian and endometrial cancer cells during cisplatin treatment

**Figure 1:**
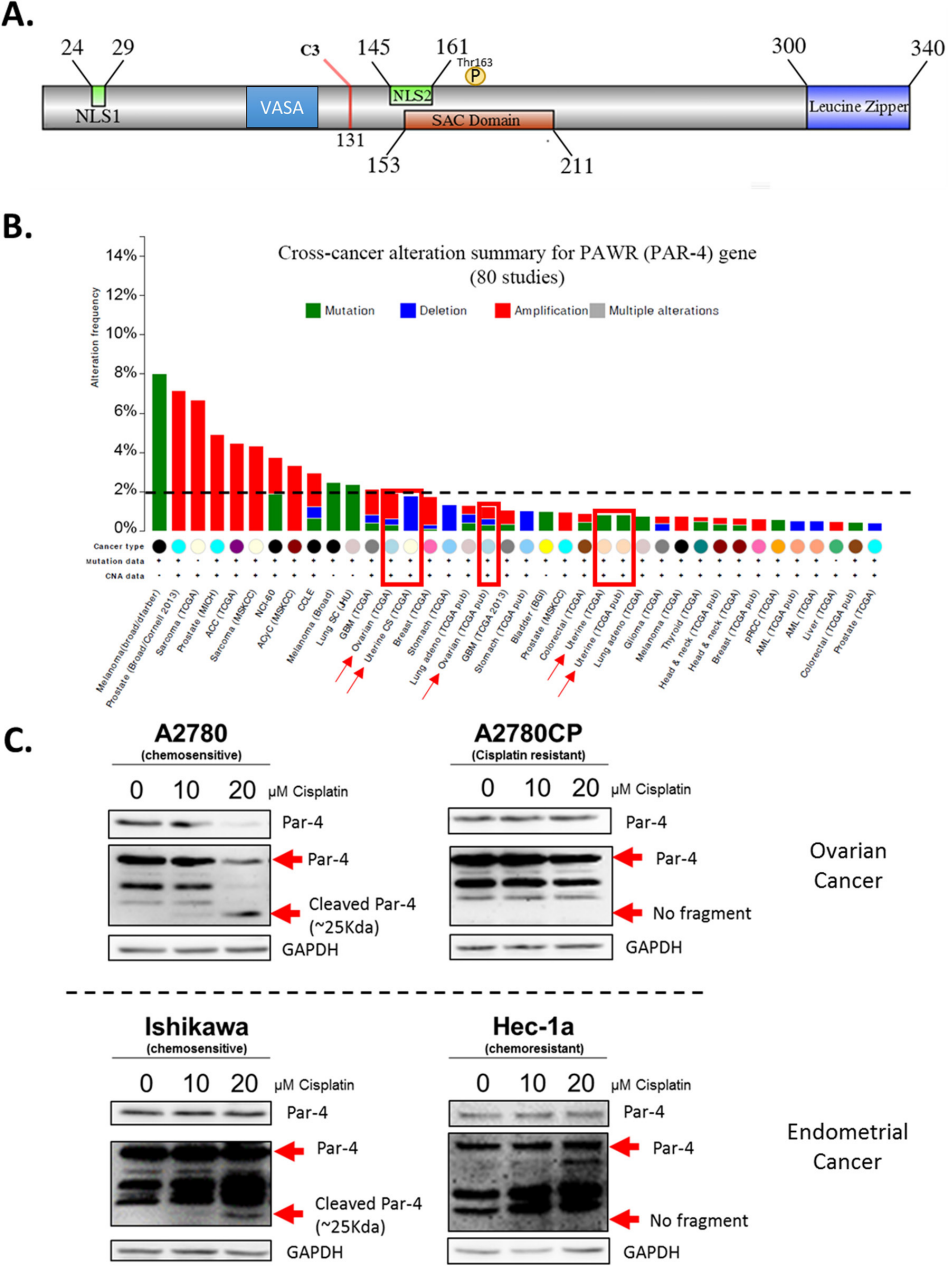
Summary of Par-4 protein and its cleavage in ovarian and endometrial cancer cells upon cisplatin treatment **A.** Schematic of Par-4 domains including the caspase-3 cleavage site location. **B.** Histogram presenting the low level of alteration of Par-4 gene (PAWR) occurring in various cancer cell lines. **C.** Ovarian cancer cell lines (A2780-A2780CP) and endometrial cancer cell lines (Ishikawa and Hec-1a) were treated with 10-20μM Cisplatin for 24h. The level of Par-4 and its cleaved fragment were determined in treated cells using western blot analysis. GAPDH was used as a loading control. Results shown are representative of three independent experiments.

Par-4 possesses various domain including the nuclear localization sequence (NLS1&2), the VASA domain for ubiquitination by Fbxo45, the selective for apoptosis in cancer cells domain (SAC) and a leucine zipper domain (Figure 1A).

A characteristic to consider for cancer therapy is the level of alteration the protein of interest possesses. In the case of Par-4, the protein is scarcely ever suppressed, nor mutated. Literature and data from cbioportal.org indicates that less than 2.5% of all cancer cases have a mutation or suppression of the Par-4 gene, excluding melanoma. This rate is even lower (<1%) in ovarian and endometrial cancers (Figure 1B) [28]. This low alteration rate combined with the tumour suppressor characteristic of Par-4 are favourable for consideration as a molecular target for cancer therapy.

We have previously demonstrated that upon cisplatin treatment, Par-4 is cleaved by caspase-3 at D132 and subsequently generates a 25kDa fragment, probed with Par-4 antibody, that we named cl-Par-4 (Figure 1A) [14]. We hence decided to evaluate if the same effect would be observed in different ovarian (A2780, A2780CP) and endometrial (Ishikawa, Hec-1a) cancer cells. Interestingly, cl-Par-4 was present in a dose-dependent manner only in chemosensitive cell lines (Ishikawa and A2780) treated with cisplatin (Figure 1C). Indeed, no additional band representing cl-Par-4 was visible at approximately 25kDa in the chemoresistant cell lines (Hec-1a and A2780CP) after cisplatin treatment (Figure 1C). These findings indicate that cancer cells from both ovarian and endometrial tissues can also cleave Par-4 and that the chemoresistance status of the cell line plays a role in the presence of cl-Par-4 upon cisplatin treatment.

### Cleaved-Par-4 is stabilized by post-translational mechanisms upon cisplatin treatment

**Figure 2:**
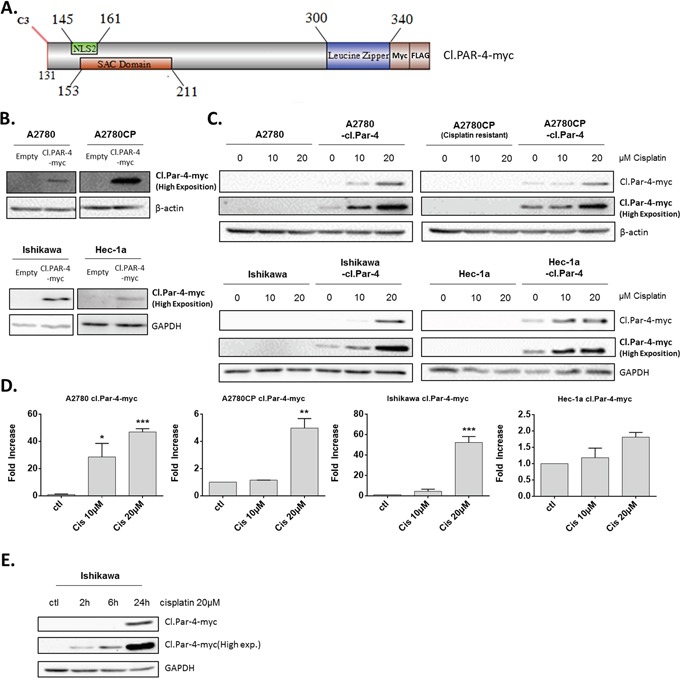
Cleaved-Par-4 protein is stabilized upon cisplatin treatment **A.** Schematic of cl-Par-4 transgene and its domains for the production of stable clones, used throughout the manuscript. **B.** Ovarian and endometrial cancer cells were stably transduced with cl-Par-4-myc plasmid using lentiviral particles. **C-D.** Cl-Par-4 cancer cell lines were treated with 10-20μM Cisplatin for 24h. **E.** Ishikawa Cl-Par-4 cancer cell lines were treated with 20μM Cisplatin for 2h, 6h or 24h. The protein level of cl-Par-4-myc was determined in treated cells using western blot analysis. β-Actin or GAPDH were used as a loading control. Results shown are representative of three independent experiments. Results are mean ± S.E.M. of three independent experiments. *=P<0.05; **=P<0.01 and ***=P<0.001 when compared with corresponding mock-treated cells.

To better explore the regulation of cl-Par-4 in ovarian and endometrial cancer cells, we used a lentiviral plasmid containing the cl-Par-4 sequence with the addition of myc-tag and FLAG (DDK) at the 3′-end (Figure 2A). Using this newly constructed lentiviral plasmid, we infected both ovarian (A2780, A2780CP) and endometrial (Ishikawa, Hec-1a) cancer cells, followed by a five-day antibiotic selection to obtain stables clones exhibiting constitutive cl-Par-4 expression (Figure 2B). Intriguingly, a very high exposition was required to see the protein levels of cl-Par-4 by Western blot indicating the levels were relatively low considering the constitutive expression of the transgene. This weak expression was observed in all four ovarian and endometrial cancer cell lines, which indicated a potential negative regulatory mechanism targeting the cl-Par-4 protein (Figure 2B). We then treated all four cell lines expressing cl-Par-4 with incremental doses of cisplatin (10-20μM) and witnessed an unequivocal dose-dependent increase in cl-Par-4 levels (Figure 2C). The effect was also related to the chemoresistance status of cell lines: chemosensitive cancer cells exhibited a dramatic 50 fold increase in cl-Par-4 levels relative to baseline conditions, while chemoresistant cancer cells showed a modest increase, with values for fold change ranging from 2 to 5 (Figure 2C-2D). These results are similar to those observed for endogenous cl-Par-4 with regards to the chemoresistance status of cell lines (Figure 1), but also reveal that cl-Par-4 accumulation is not only the result of a caspase-3-dependent cleavage and may also involve other regulatory mechanisms.

Finally, we treated Ishikawa with cisplatin at different time points (2h, 6h and 24h) to determine if cl-Par-4 accumulation was detectable before 24h. The results clearly show that cl.Par-4 levels are increased as soon as 2h after treatment with 20μM cisplatin indicating that this regulation is rapid and likely mediated by post-translational mechanisms (Figure 2E).

### Cleaved-Par-4 subcellular localization in ovarian and endometrial cancer cell lines

**Figure 3:**
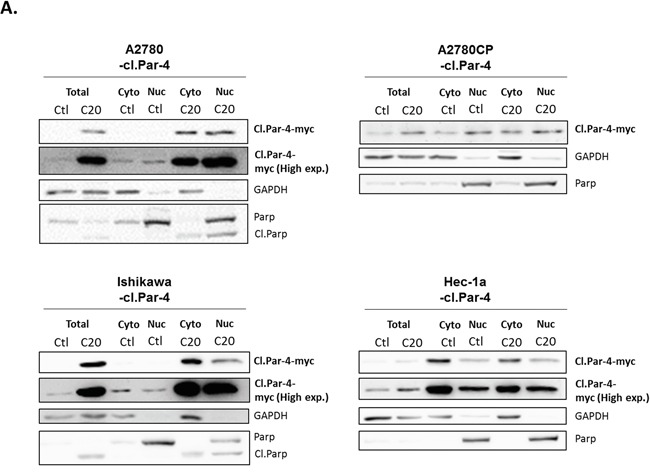
Cleaved-Par-4 localization in ovarian and endometrial cancer cell lines Cl-Par-4 cancer cell lines were treated with 20μM Cisplatin for 24h. **A.** Cytosolic/nuclear cell fractionation was done and the protein level of cl-Par-4-myc was determined in treated cells using western blot analysis. GAPDH and PARP were used as cytosolic and nucleus loading control respectively. Results shown are representative of three independent experiments.

Since full-length Par-4 can be found in both the cytoplasm and nucleus of cancer cells, we questioned if cl-Par-4 was also localized in both compartments and if a translocation would occur upon cisplatin treatment to increase the level of cl-Par-4 as previously observed. Using subcellular fractionation and western blot, we observed a pattern where cl-Par-4 was localized in both the cytoplasm and nucleus in ovarian and endometrial cancer cell lines in presence or absence of cisplatin (Figure 3A). No apparent nuclear translocation occurred upon cisplatin treatment. These results indicated that the localization of cl-Par-4 was not implicated in the post-translational regulation observed when using cisplatin.

### Cleaved-Par-4 protein level is decreased by the proteasome

**Figure 4:**
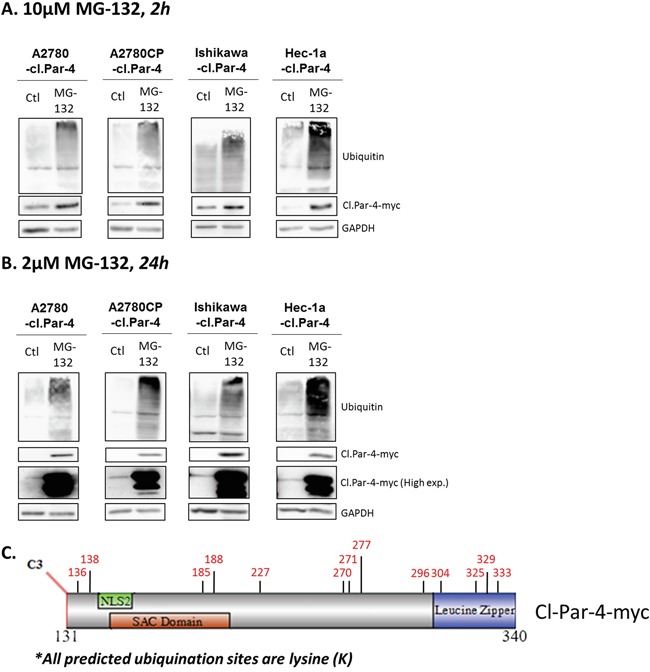
Proteasome negative regulation of cleaved-Par-4 **A-B.** Cl-Par-4 cancer cells were treated with either 10μM MG-132 for 2h or 2μM MG-132 for 24h. **C.** Schematic of cl-Par-4 transgene and the predicted ubiquitination sites from bioinformatic analysis.

Considering that full-length Par-4 is regulated by various post-translational mechanisms including ubiquitination and proteasome-dependent degradation, we assessed whether cl-Par-4 could be regulated in a similar manner by treating ovarian and endometrial cancer cells with a proteasome inhibitor, MG-132 (Figure 4A-4B). MG-132 inhibits the degradation of ubiquitinated proteins but is also known to be able to induce apoptosis [39, 40]. To prevent this undesired effect, we first used a standard 10μM dose of MG-132, but for a short period (2h) and a considerable increase in cl-Par-4 levels was observed (Figure 4A). An even more significant increase was detected using a lower dose (2μM) for 24h (Figure 4B).

Subsequently, we performed bioinformatics analyses using online databases to verify if algorithms could predict any potential ubiquitination sites (Figure 4C). First, we used the phosphosite plus database and found a potential site at K333 located on leucine zipper domain [41, 42]. Phosphosite plus supports this prediction with six different manuscripts which predicted the K333 site using proteomic discovery-mode mass spectrometry, thus strengthening the likelihood of a true ubiquitination site [41, 42]. In parallel, we used three additional bioinformatics tools (Ubiprober, Ubpred, BDM-PUB) and identified 13 candidate ubiquitination sites, including the K333 site previously predicted by discovery-mode mass spectrometry (Table 1) [43–45]. By looking at the position of each predicted site, we found that K185 and K188 are located within the SAC domain while K305, K325, K329 and K333 are located within the leucine zipper domain (Table 1) (Figure 4C). Altogether, these results suggest that cl-Par-4 is ubiquitinated.

**Table 1:** Prediction of ubiquitination sites for cleaved-PAR-4

Position*	Sequence	Database ressource
136	EPDGVPE**-K-**GKSSGPS	BDM-PUB; Ubpred; Ubiprober
138	DGVPEKG**-K-**SSGPSAR	BDM-PUB; Ubpred; Ubiprober
185	EDDEAGQ**-K-**ERKREDA	BDM-PUB; Ubpred
188	EAGQKER**-K-**REDAITQ	BDM-PUB; Ubpred
227	RTVSGRY**-K-**STTSVSE	BDM-PUB; Ubpred
270	VSSSTLE**-K-**KIEDLEK	BDM-PUB; Ubpred
271	SSSTLEK**-K-**IEDLEKE	BDM-PUB; Ubpred
277	KKIEDLE-**K**-EVVRERQ	Ubpred; Ubiprober
296	LVRLMQD-**K**-EEMIGKL	Ubpred; Ubiprober
304	EEMIGKL-**K**-EEIDLLN	Ubpred; Ubiprober
325	EDENEQL-**K**-QENKTLL	Ubpred; Ubiprober
329	EQLKQEN-**K**-TLLKVVG	Ubpred; Ubiprober
333	QENKTLL**-K-**VVGQLTR	BDM-PUB; Ubiprober; Phosphosite plus

Ubiquitination sites were predicted using four bioinformatics database (BDM-Pub; Ubpred; Ubiprober and Phosphosite plus). 13 potential sites of ubiquitination were predicted considering they were provided by at least two bioinformatics tools. The position indicated in the table is based on the full length of Par-4 sequence (NP_002574.2). All sites are located on a Lysine (K).

* Position is based on full length Par-4 sequence

**Figure 5:**
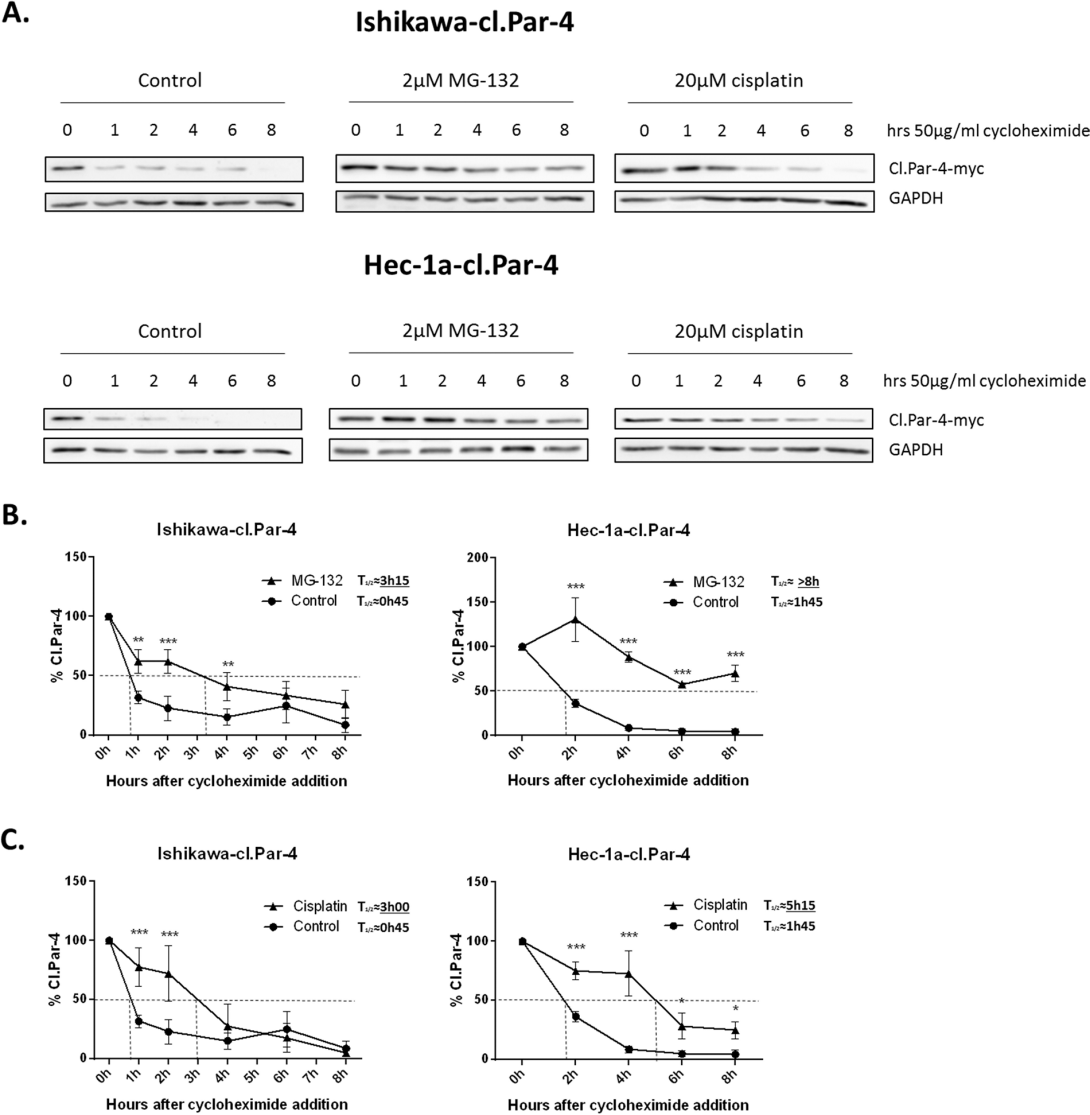
Cleaved-Par-4 protein stability **A.** Endometrial cl-Par-4 cancer cells were treated or not with either 2μM MG-132 or 20μM for 24h and 50μg/ml cycloheximide was added 1-8h before the end of treatment. The protein level of cl-Par-4-myc was determined in treated cells using western blot analysis. GAPDH was used as a loading control. Results shown are representative of three independent experiments. **B.** Graph representing cl-Par-4 protein stability when using cycloheximide in combination or not with MG-132. **C.** Graph representing cl-Par-4 protein stability when using cycloheximide in combination or not with cisplatin. Results are mean ± S.E.M. of three independent experiments. *=P<0.05, **=P<0.01 and ***=P<0.001 when compared with corresponding mock-treated cells.

Ubquitination and proteasome degradation often plays a critical role in protein stability. Considering the previous observations with the different compounds, we wanted to know if they were related to the protein stability of cl-Par-4. To determine whether cisplatin-induced cl-Par-4 accumulation was solely the result of increased protein stability or also the result of increased translation, we treated ovarian and endometrial cancers cells with cycloheximide, an inhibitor of protein biosynthesis, at various time points alone or in combination with cisplatin or MG-132 as a positive control (Figure 5). Firstly, cl-Par-4 protein half-life was measured under normal condition in both Ishikawa and Hec-1a cells and 1h30 was approximately the time required after cycloheximide addition to obtain 50% of remaining protein (Figure 5). Combining cycloheximide with MG-132 abrogate cl-Par-4 accumulation supporting the post-translational stabilization of cl-Par-4 by the proteasome (Figure 5A-5B). In the presence of MG-132, half-life of cl-Par-4 significantly increased in Ishikawa cells (approximately 3h15) and not yet attained in Hec-1a cells after 8h treatment with cycloheximide (Figure 5B). Combining cycloheximide with cisplatin did abrogate cl-Par-4 accumulation in a similar manner as observed with MG-132 (Figure 5C). In both Ishikawa and Hec-1a cells, the half-life of cl-Par-4 was similarly increased with MG-132, as with cisplatin, hence supporting the post-translational stabilization of cl-Par-4 upon cisplatin treatment (Figure 5A & 5C).

### PI3K and MAPK pathways are involved in the regulation of cleaved-Par-4 levels

We next investigated whether well-established pro-survival PI3K and MAPK pathways in ovarian and endometrial cancers could be involved in the post-translational regulation of cl-Par-4.

**Figure 6:**
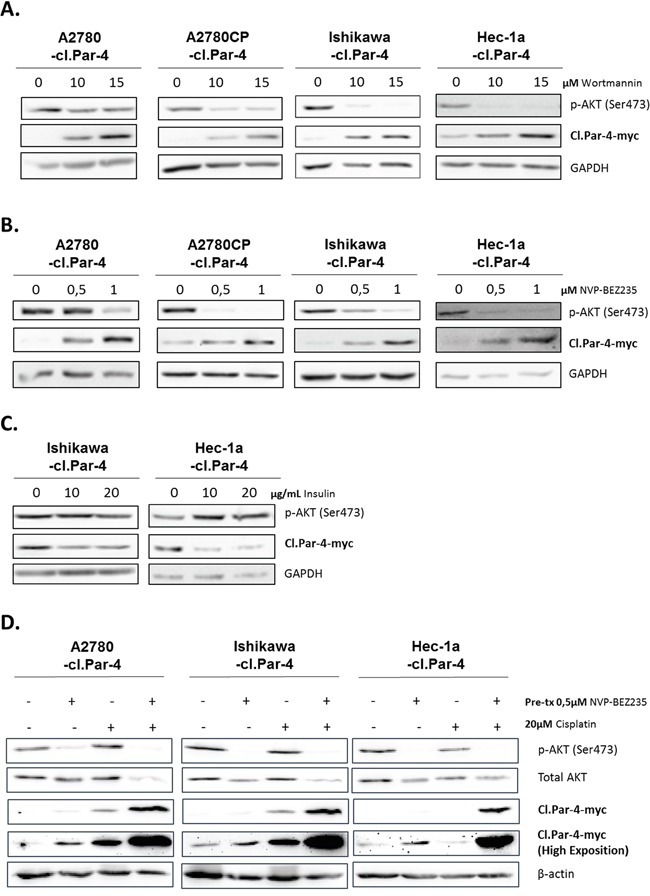
PI3K pathway decreases cleaved-Par-4-myc protein **A.** Cl-Par-4 cancer cells were treated with 10 or 15μM PI3K inhibitor (Wortmannin) for 24h. **B.** Cl-Par-4 cancer cells were treated with 0,5 or 1μM clinical PI3K inhibitor (NVP-BEZ235) for 24h. **C.** Endometrial cancers cells were treated with 10 or 20μg/mL of insulin to induce PI3K activity for 30 min. **D.** Cl-Par-4 cancer cells were pre-treated with 0,5μM clinical PI3K inhibitor (NVP-BEZ235) for 24h followed by a 24h treatment of 20μM cisplatin. The levels of p-AKT (Ser473), total AKT and cl-Par-4-myc were determined in treated cells using western blot analysis. GAPDH or β-actin were used as loading controls. Results shown are representative of three independent experiments.

We first looked at the PI3K pathway using the common PI3K inhibitor Wortmannin at various doses and observed a significant dose-dependent increase in cl-Par-4 protein levels (Figure 6A). We pursued experimentations with the use of a clinical PI3K inhibitor, NVP-BEZ-235 that positively supported the previous results and observed a significant increase of cl-Par-4, again in a dose-dependent manner (Figure 6B). To further support the role of PI3K signaling in the process, we used insulin to activate the PI3K pathway and assessed whether this would cause the down-regulation of cl-Par-4. In line with our hypothesis, cl-PAR-4 level was decreased within 30 min of insulin treatment (Figure 6C). We also examined if the increase of cl-Par-4 previously observed with cisplatin treatments was PI3K-dependent. In order to answer this question, we pre-treated cancer cells with NVP-BEZ235 to initially inhibit PI3K activity in cancer cells. Following the pre-treatment, cancer cells were treated with 20μM cisplatin for 24h. Combining both NVP-BEZ235 with cisplatin increased cl-PAR-4 in a synergetic manner in all three cancer cell lines indicating that the positive regulation of cl-PAR-4 previously observed with cisplatin alone was not solely PI3K-dependent (Figure 6D). Indeed, if PI3K was solely responsible for the protein increase of cl-Par-4 observed with cisplatin, the protein level when comparing the pre-treatment with NVP-BEZ235 versus its combination with cisplatin would not be significantly different, which is not the case in our experiment. PI3K inhibition, thus, contribute to the effect observed with cisplatin but is not the only responsible protein.

AKT is one of the main downstream targets of PI3K and we wondered if this protein was implicated in the regulation observed with PI3K inhibition and cl-Par-4. To do so, we used three different pan-AKT inhibitors (MK-2206, AZD5363 and Perifosine). Both MK-2206 and Perifosine inhibitors are known to strongly reduce the phosphorylation level of AKT and its activity while AZD5363 is known to inhibit the phosphorylation of AKT downstream substrates [46–48]. The obtained results suggest that inhibition of AKT activity does not regulate cl-Par-4 as we observed with PI3K (Figure S1). Indeed, the regulation observed with AKT inhibitors is the opposite of what we previously observed with PI3K inhibitors. Consequently, this indicate that PI3K downregulates cl-Par-4, independently of AKT.

**Figure 7:**
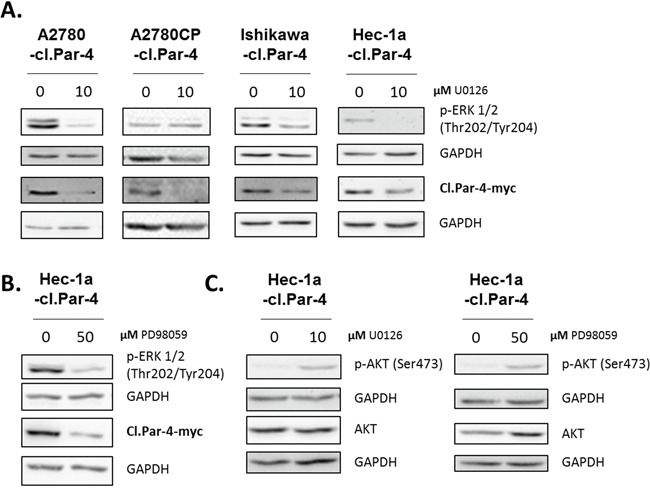
MAPK pathway increases cleaved-Par-4 protein **A-C.** Cl-Par-4 cancer cells were treated with either 10μM MAPK inhibitor (U0126) or 50μM MAPK inhibitor (PD98059) for 24h. The levels of p-ERK 1/2 (Thr202/Tyr204), p-AKT (Ser473), AKT and cl-Par-4-myc were determined in treated cells using western blot analysis. GAPDH was used as a loading control. Results shown are representative of three independent experiments.

Next, we investigated the MAPK pathway using U0126, an inhibitor of MEK1 and MEK2 kinases. MAPK inhibition caused the down-regulation of cl-Par-4 in both ovarian and endometrial cancer cell lines (Figure 7A). We observed similar effect with the MAPK pathway inhibitor PD98059 in Hec-1a endometrial cancer cells (Figure 7B). These findings are the exact opposite of what we obtained with PI3K inhibitors, and could be explained by a regulatory cross-talk between PI3K and MAPK pathways. In support of this, we found that MAPK inhibition led to an increase in p-Akt (S473), a downstream target of PI3K in Hec-1a endometrial cancer cells (Figure 7C). Overall, these results demonstrate that both the PI3K and MAPK pathways can regulate cl-Par-4 differentially.

### Integrating the signaling pathways responsible for cleaved Par-4 regulation

**Figure 8:**
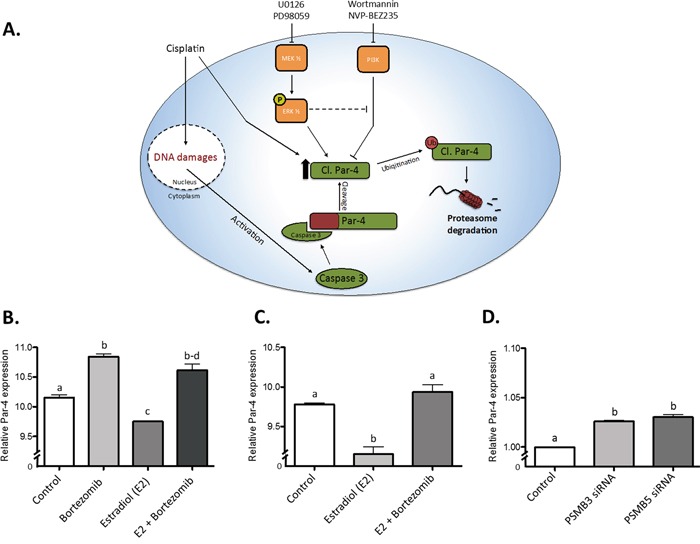
Graphical representation of the proposed model and public data integration **A.** The proposed model of regulation regarding the cleaved fragment of Par-4. (B-D) Data from two independent dataset taken from a study from Prentzel et al. (PMID 21862633). Relative Par-4 mRNA expression was used in all cases. **B.** The use of Bortezomib, a potent proteasome inhibitor, significantly increases Par-4 expression in MCF7 cells while estradiol significantly reduces it. However, the use of both reverse the negative effect of estradiol (GEO accession GDS4089). **C.** The use of estradiol in MCF7 cells significantly reduces Par-4 expression and Bortezomib treatment reverse this effect (GEO accession GDS4090). **D.** The knockdown of PSMB3 and PSMB5 (Proteasome subunits) induces a significant increase of Par-4 mRNA in MCF7 cells (GEO accession GDS4090). Results are mean ± S.E.M. of three independent experiments and P<0.05.

In light of the results shown earlier in this study, we posit that a complex signaling network, composed of multiple regulatory elements, is capable of modulating cleaved Par-4 levels. A schematic representation of the findings of our study can be found in Figure 8A. This cartoon clearly shows the intricate relationship between multiple pathways and regulatory mechanisms, all converging into the upregulation of the amount of cl-Par4 found in the cell. Interestingly, by perusing various databases and submitting them to bioinformatics analyses, we uncovered novel similar conclusions in other models [49]. MCF7 cells subjected to Bortezomib treatment, a clinically used proteasome inhibitor, exhibited significantly increased Par-4 expression; the same experiment showed a sharp and significant decrease of Par-4 expression following 17β-estradiol (E2) treatment (Figure 8B). A similar experiment from a different dataset suggested the same effect on Par-4 (Figure 8C). Finally, the knockdown of proteasome subunits PSMB3 and PSMB5 were shown to induce significantly increased Par-4 expression in MCF7 cells (Figure 8D). Taken together, these results suggest a complicated but certain relationship between proteasome activity, E2 signaling and Par-4 expression.

## DISCUSSION

Par-4 is known for being a complex protein associated with multiple pro-apoptotic mechanisms and regulated by several post-translational modifications [9, 10]. Indeed, like previously introduced, Par-4 can be modified through various processes including phosphorylation, ubiquitination and proteolytic cleavage.

In the present manuscript, we focused on the ~25kDa cleaved form of Par-4 which has been previously reported as a product of Par-4 following its cleavage by caspase-8 and caspase-3 under apoptotic stimuli [14, 37]. We found that in ovarian and endometrial cancer cells, cl-Par-4 undergoes rapid degradation by the proteasome in baseline conditions but is stabilized upon cisplatin treatment. Furthermore, we found out that PI3K and MAPK pathways were involved in the regulation of cl-Par-4 stability using different inhibitors.

We and others have shown that Par-4 is cleaved by caspases 3-8 under apoptotic circumstances [14, 37]. As observed in the current findings, the cleavage did not occur in chemoresistant cancer cells probably because pro-caspases, which did not undergo cleavage, are required to produce the cl-Par-4 fragment. However, we also discovered an astonishing regulation of cl-Par-4, independently of the full-length cleavage, when treated with cisplatin. The regulation observed was dependent on chemoresistance status, in both ovarian and endometrial tissue, indicating that mechanisms of resistance also play a role in stabilization of Par-4. This stabilization observed with cisplatin could be related to the proteasome considering we were able to stabilize the protein level similarly and efficiently using either MG-132 or cisplatin when used in combination with cycloheximide to inhibit *de novo* protein synthesis. Cisplatin stabilizing different proteins via a post-translational mechanism, just like we observed with cl-Par-4, is not something new. Indeed, in the literature, many papers report cisplatin to be implicated in the stabilization of various proteins via the ubiquitin-proteasome, a mechanism required to induce apoptosis efficiently with cisplatin. Interestingly, mechanisms of chemoresistance are also related to these proteins and their ease of stabilization by cisplatin and the proteasome [50–56]. Furthermore, we observed that Hec-1a seems to have a lower level of cl-Par-4 protein localized in the nucleus when compared with the other cell lines. This difference does not seem to be major but could also be linked to the high chemoresistance profile of the cancer cell line and the possibility of regulation by the proteasome.

Localization of the Par-4 protein has been shown to play an important role in its cellular functions. Actually, various papers reported that the translocation of Par-4 to the nucleus was needed to induce apoptosis and stimulate the transcription of diverse genes [9, 57, 58]. Concerning cl-Par-4, it was previously demonstrated that this product was localized in both cytosol/nucleus with a modest trend for nuclear enrichment [14, 37]. In the present study, we showed that cl-Par-4 was localized in both compartments with slight differences between the different cell lines used. The observed localization from our experiments could be related to tissue specificity (endometrial and ovarian), whereas the other manuscripts investigated the commonly used HeLa cervical cancer cells or HEK293 human embryonic kidney cells [14, 37]. It is also interesting to note that in estrogen-dependent cancers (ovarian and endometrium), other studies have shown Par-4 being mainly localized in the cytoplasm [7].

Our results implicate proteasome degradation as a major regulator of cl-Par-4 stability. This interaction with the proteasome seems to play an important role on the stability of the cleaved protein when looking at the highly increased half-life of the protein when using cycloheximide in combination with proteasome inhibitor, MG-132. Interestingly, a recent study has shown that Par-4 is ubiquitinated by Fbxo45 leading to Par-4 proteasomal degradation [35]. However, cl-Par-4 is devoid of this ubiquitination site: the VASA-like region where Fbxo45 binds on Par-4 is located before amino acid 120, which is upstream of the caspase-3 cleavage site at D131 (Figure 1A) [14]. To our knowledge, no other sites of ubiquitination have been found in cl-Par-4.

Interaction of the PI3K pathway and Par-4 is already reported in the literature. Indeed, AKT1, a downstream target of PI3K, can bind and phosphorylate Par-4 to inhibit its activity and prevent translocation to the nucleus. Par-4 activity is also linked to PTEN tumor suppressor activity since PTEN is a negative regulator of the PI3K pathway [24]. Due to the high mutation rate of PTEN in ovarian and endometrial cancers, PI3K is unrestrained, leading to a high AKT1 phosphorylation and subsequent inhibition of Par-4 activity which is related to chemoresistance [28, 29, 59, 60]. In the case of cl-Par-4, Wortmannin and NVP-BEZ235 inhibits PI3K upstream and downstream targets should be considered. We decided to check if AKT, one of the main target of PI3K, was responsible for the regulation observed with PI3K inhibitors and the results obtained showed that PI3K downregulates cl-PAR-4 independently of AKT. PI3K can regulate various proteins independently of AKT and one possible candidate for the regulation observed with PI3K could be PDK1 kinase and its downstream targets [61]. Par-4 has been shown to downregulate the kinase PDK1, a protein upstream of AKT and downstream of PI3K [62]. Through PDK1, independently of AKT, the proteins SGKs can be regulated and are, in part, responsible for cell survival, proliferation, and growth. SGKs can block apoptosis by inhibiting FOXO proteins just like AKT [61, 63–65]. FOXO3a have previously been observed in relation with PAR-4 by allowing an increase of the transcription of its gene (PAWR) and thus, allowing an increase of apoptosis [66]. It is worth noting, however, that these interactions have been associated with AKT and transcription, which are not the case in our observations here where we observed, instead, post-transcriptional regulation and AKT independent regulation. Interestingly, in our experiments, we are using the cleaved fragment of PAR-4 in feminine cancer models. PKC is another downstream target of PDK1, independent of AKT, which could be responsible for the obtained results [64, 67]. Binding between PAR-4 and PKC zeta/lambda have previously been observed in NIH-3T3 fibroblasts and could also be related to our observations with the PI3k inhibitors. The binding of PAR-4 with PKC inhibits the pro-survival activities of theses kinases (PKC zeta and lambda) and thus increasing PAR-4 pro-apoptotic activity [68]. Cisplatin is known for being able to downregulate PI3K downstream targets but resistance to this drug is also caused in part by a downregulation of Par-4 and an increase of the PI3K pathway [33, 71, 72]. Using PI3K inhibitors to increase Par-4, leading to an increase of cl-Par-4, in combination with a chemotherapeutic drug such as cisplatin is an interesting avenue to overcome cancer cells chemoresistance.

Par-4 expression is known for being down-regulated by the Ras oncogene in different models [18, 73, 74]. Indeed, Ras can regulate many downstream targets including Raf, which in turn is involved in the activation of the MAPK pathway [18, 74]. It has been demonstrated that inhibition of MAPK using different inhibitors, including U0126, can restore PAR-4 protein level [18, 74]. Another article indicated a similar effect where MCF-7 breast cancer cells overexpressing Par-4 showed a reduced level of phosphorylation for ERK 1/2 [75]. Ras can also activate the PI3K pathway, however, studies have shown that Ras regulation of Par-4 was not dependent on this survival pathway [18, 74]. In the present study, we observed a different effect where the cleaved fragment of Par-4 was downregulated when using MAPK inhibitors. Considering cl-Par-4 is a sub-product of the protein Par-4, the mechanism of regulation could be slightly different. The type of tissue, here ovary and endometrium, could also influence the effect on cl-Par-4. In addition, inhibition of the MAPK pathway can have an impact on the PI3K pathway by regulating some of its targets such as p-AKT and PDK1 [76, 77]. While inhibiting MAPK, we observed an increase of p-AKT (S473) but a decrease of cl-Par-4; PI3K inhibition, however, presented the opposite effect with a sharp increase in cl-Par-4 and largely reduced p-AKT. This suggest that a cross-talk between PI3K and MAPK pathways could explain these opposite but possibly intertwined effect [77].

As introduced, not much has yet been studied concerning Par-4 in endometrial and ovarian cancer tissues. Endometrium and ovary are known for being hormone-dependent tissues and, interestingly, hormones have an important role in Par-4 regulation. Indeed, as demonstrated by our analysis using bioinformatics datasets, estradiol negatively regulates Par-4 mRNA in MCF-7 breast cancer cell [49]. Another manuscript also stated the same negative effect on Par-4 in MCF-7 breast cancer cells treated with estradiol [78]. Likewise with the bioinformatics dataset, we also observed that the negative effect of estradiol on Par-4 could be canceled using a proteasome inhibitor, then leading to an increase of Par-4 in a way similar to what we observed in the case of cl-Par-4 throughout this manuscript in the context of proteasome inhibition. Very interestingly, the knockdown of proteasome subunits also yield a modest but significant increase in Par-4 mRNA; these results, combined with the results proposed in this study, show that proteasomal degradation of Par-4 is only partly responsible for cl-Par-4 control. The fact that both protein and mRNA levels are modulated through some form of proteasome regulation suggest that a protein under the influence of proteasomal degradation is capable of regulating Par-4 transcription. Considering the high turnover of estrogen receptors and the possible implication of the proteasome in regulating estrogen receptor stability and activation, we find this future avenue of research very compelling [79, 80].

Additionally, in prostate cancer, an androgen-dependent tissue, it was demonstrated that Par-4 was efficient for inducing apoptosis only in hormone-independent cancer cells [7, 25]. Both androgen and estrogen are known for being able to activate the PI3K pathway [81, 82]. Following the logic of the previous observation with estrogen in MCF-7 breast cancer cells, the regulation observed with Par-4 could be PI3K-dependent. Indeed, we observed a regulation of cl-Par-4 using PI3K inhibitors. The hormones presents in our endometrial and ovarian cancer models could be involved in the instability of our protein, cl-Par-4, via the PI3K network. Considering the vast amounts of mechanisms hormones are involved with, it would be relevant to further investigate Par-4 and cl-Par-4 functions in hormone-dependent cancer models such as endometrial and ovarian cancers.

All these findings demonstrate undiscovered regulation mechanisms of Par-4. The observed mechanisms of regulation justify further studies addressing the biological significance of Par-4 regulation in relation to cancer chemosensitivity.

## MATERIALS AND METHODS

### Cell culture

Human endometrial cancer cell lines Ishikawa and Hec-1a were kindly provided by Dr. Sylvie Mader (Université de Montréal, Montréal, Canada); A2780 and A2780CP (Cisplatin resistant) were kindly provided by Dr. G. Peter Raaphorst (Ottawa regional cancer center, Ottawa, Canada). Hec-1a cell line was maintained in McCoy's 5A Medium containing 5% bovine growth serum and 50 μg/ml gentamycin; Ishikawa, A2780 and A2780CP cell lines were maintained in DMEM-F12 medium containing 2% bovine growth serum and 50 μg/ml gentamycin. The cells were maintained at 37°C with 5% CO_2_.

### Lentiviral transfection

Lentiviral particles were produced using the lenti-X HTX packaging system and HEK-293T cells from Clontech laboratories (Mountain View, CA, USA). The cl-Par-4-myc-DDK plasmid was constructed from a pLVX-puro backbone plasmid (Clontech laboratories, Mountain View, CA, USA). The cl-Par-4 portion of the plasmid was added using the InFusion cloning system from Clontech (Mountain View, CA, USA). The cl-Par-4 fragment also contains a myc-tag sequence as well as a DDK (Flag) sequence at 3′ -end. Ovarian cancer cells (A2780 and A2780CP) and endometrial cancer cells (Ishikawa and Hec-1a) were then infected with supernatant containing lentiviral particles of either empty pLVX-puro or pLVX-cl-Par-4-myc-DDK plasmid for 24h. Media was then replaced and cells were let to recover for 24h. Antibiotic selection of transduced cells were then done using puromycin (0,75μg/ml for Hec-1a and 0,5μg/ml for A2780, A2780CP and Ishikawa) for five days. The whole pool containing the resistant cells were then used for further experimentations.

### Antibodies and reagents

All primary antibodies were obtained from Cell Signaling Technology (Bervely, MA, USA) except for the loading controls GAPDH (Abcam, Cambridge, MA, USA) and β-actin (Sigma-Aldrich, St-Louis, MO, USA). Secondary antibodies, HRP-conjugated goat anti-rabbit was from Bio-Rad Laboratories (Mississauga, ON, Canada). Cisplatin, 17β-estradiol, Cycloheximide, Wortmannin, Insulin and MG-132 were purchased from Sigma-Aldrich (St-Louis, MO, USA), NVP-BEZ235 was purchased from Cayman Chemical (Ann Arbor, MI, USA), MK-2206, Perifosine and AZD5364 were purchased from Selleck Chemicals (Houston, TX, USA), U0126 and PD98059 were purchased from Cell Signaling Technology (Bervely, MA, USA).

### Western blot

Treated cells were washed with PBS and submitted to lysis in cold RIPA buffer containing protease and phosphatase inhibitors (Roche Applied Science, Laval, QC, Canada) followed by three freeze-thaw cycles. Equal amounts of cell lysates, determined using Bio-Rad DC protein assay (Mississauga, ON, Canada), were separated by SDS-Page polyacrylamide gels (8-15%) and then transferred onto nitrocellulose membranes (Bio-Rad, Mississauga, ON, Canada). Membranes were blocked in 5% milk, PBS 1X, 0.06% Tween 20 for 1 h at room temperature, probed with primary antibody, washed in PBS 1X, 0.06% Tween 20, and incubated with horseradish peroxidase-conjugated secondary antibody (Bio-Rad, Mississauga, ON, Canada). Detection was performed using SuperSignal West Femto™ substrate (Thermo Fisher Scientific, Nepean, ON, Canada), as described by the manufacturer using UVP bioimaging systems. Densitometry was done using either Quantity One software version 4.6.9 (Bio-rad, Mississauga, ON, Canada) or ImageJ software 1.50B [83].

### Subcellular fractionation

NE-PER Nuclear and Cytoplasmic Extraction Reagent (Thermo Fisher Scientific, Nepean, ON, Canada) was used according to the manufacturer's instructions. Cytoplasmic proteins were collected using CERI&II reagents while proteins from the nucleus were collected using NER reagent. GAPDH was used as a loading/purity control for cytoplasmic proteins while PARP was used for nuclear proteins.

### Statistical analyses

The data were subjected to one-way or two-way analysis of variance (One-way/Two-way ANOVA) using PRISM software (version 6.00; GraphPad, San Diego, CA). Differences between experimental groups were determined by the Tukey's test. Statistical significance was accepted when p < 0.05.

## SUPPLEMENTARY FIGURE



## References

[1] American Cancer Society (2015). Cancer Facts & Figures 2015.

[2] Canadian Cancer Society's Advisory Committee on Cancer Statistics (2015). Canadian Cancer Statistics.

[3] Ferlay J, Steliarova-Foucher E, Lortet-Tieulent J, Rosso S, Coebergh JWW, Comber H, Forman D, Bray F (2013). Cancer incidence and mortality patterns in Europe: Estimates for 40 countries in 2012. European Journal of Cancer.

[4] Ahmad N, Kumar R (2011). Steroid hormone receptors in cancer development: A target for cancer therapeutics. Cancer Letters.

[5] Ali AY, Farrand L, Kim JY, Byun S, Suh J-Y, Lee HJ, Tsang BK (2012). Molecular determinants of ovarian cancer chemoresistance: new insights into an old conundrum. Annals of the New York Academy of Sciences.

[6] Chaudhry P, Asselin E (2009). Resistance to chemotherapy and hormone therapy in endometrial cancer. Endocr Relat Cancer.

[7] El-Guendy N, Zhao Y, Gurumurthy S, Burikhanov R, Rangnekar VM (2003). Identification of a Unique Core Domain of Par-4 Sufficient for Selective Apoptosis Induction in Cancer Cells. Molecular and Cellular Biology.

[8] Shrestha-Bhattarai T, Rangnekar VM (2010). Cancer-selective apoptotic effects of extracellular and intracellular Par-4. Oncogene.

[9] Irby RB, Kline CL (2013). Par-4 as a potential target for cancer therapy. Expert Opinion on Therapeutic Targets.

[10] Hebbar N, Shrestha-Bhattarai T, Rangnekar V, Grimm S (2014). Cancer-Selective Apoptosis by Tumor Suppressor Par-4.

[11] Uhlén M, Fagerberg L, Hallström BM, Lindskog C, Oksvold P, Mardinoglu A, Sivertsson Å, Kampf C, Sjöstedt E, Asplund A, Olsson I, Edlund K, Lundberg E, Navani S, Szigyarto CA-K, Odeberg J (2015). Tissue-based map of the human proteome. Science.

[12] García-Cao I, Duran A, Collado M, Carrascosa MJ, Martín-Caballero J, Flores JM, Diaz-Meco MT, Moscat J, Serrano M (2005). Tumour-suppression activity of the proapoptotic regulator Par4. EMBO Reports.

[13] Chaudhry P, Fabi F, Singh M, Parent S, Leblanc V, Asselin E (2014). Prostate apoptosis response-4 mediates TGF-[beta]-induced epithelial-to-mesenchymal transition. Cell Death Dis.

[14] Chaudhry P, Singh M, Parent S, Asselin E (2012). Prostate Apoptosis Response 4 (Par-4), a Novel Substrate of Caspase-3 during Apoptosis Activation. Molecular and Cellular Biology.

[15] Moreno-Bueno G, Fernandez-Marcos PJ, Collado M, Tendero MJ, Rodriguez-Pinilla SM, Garcia-Cao I, Hardisson D, Diaz-Meco MT, Moscat J, Serrano M, Palacios J (2007). Inactivation of the Candidate Tumor Suppressor Par-4 in Endometrial Cancer. Cancer Research.

[16] Saegusa M, Hashimura M, Kuwata T, Okayasu I (2010). Transcriptional regulation of pro-apoptotic Par-4 by NF-κB/p65 and its function in controlling cell kinetics during early events in endometrial tumourigenesis. The Journal of Pathology.

[17] Meynier S, Kramer M, Ribaux P, Tille JC, Delie F, Petignat P, Cohen M (2015). Role of PAR-4 in ovarian cancer. Oncotarget.

[18] Pruitt K, Ülkü AS, Frantz K, Rojas RJ, Muniz-Medina VM, Rangnekar VM, Der CJ, Shields JM (2005). Ras-mediated Loss of the Pro-apoptotic Response Protein Par-4 Is Mediated by DNA Hypermethylation through Raf-independent and Raf-dependent Signaling Cascades in Epithelial Cells. Journal of Biological Chemistry.

[19] Gonzalez I, Santana P, Gonzalez-Robayna I, Ferrer M, Morales V, Blanco F, Fanjul L (2007). Regulation of the expression of prostate apoptosis response protein 4 (Par-4) in rat granulosa cells. Apoptosis.

[20] Feng Z, Zhang J-t (2005). Long-term melatonin or 17β-estradiol supplementation alleviates oxidative stress in ovariectomized adult rats. Free Radical Biology and Medicine.

[21] Boghaert E, Sells S, Walid A, Malone P, Williams N, Weinstein M, Strange R, Rangnekar V (1997). Immunohistochemical analysis of the proapoptotic protein Par-4 in normal rat tissues. Cell Growth Differ.

[22] Gurumurthy S, Goswami A, Vasudevan KM, Rangnekar VM (2005). Phosphorylation of Par-4 by Protein Kinase A Is Critical for Apoptosis. Molecular and Cellular Biology.

[23] Azmi AS, Philip PA, Zafar SF, Sarkar FH, Mohammad RM (2010). PAR-4 as a Possible New Target for Pancreatic Cancer Therapy. Expert opinion on therapeutic targets.

[24] Goswami A, Burikhanov R, de Thonel A, Fujita N, Goswami M, Zhao Y, Eriksson JE, Tsuruo T, Rangnekar VM (2005). Binding and Phosphorylation of Par-4 by Akt Is Essential for Cancer Cell Survival. Molecular cell.

[25] Chakraborty M, Qiu SG, Vasudevan KM, Rangnekar VM (2001). Par-4 Drives Trafficking and Activation of Fas and FasL to Induce Prostate Cancer Cell Apoptosis and Tumor Regression. Cancer Research.

[26] Garcia-Cao I, Lafuente MJ, Criado LM, Diaz-Meco MT, Serrano M, Moscat J (2003). Genetic inactivation of Par4 results in hyperactivation of NF-κB and impairment of JNK and p38. EMBO Reports.

[27] Wang B-D, Kline CLB, Pastor DM, Olson TL, Frank B, Luu T, Sharma AK, Robertson G, Weirauch MT, Patierno SR, Stuart JM, Irby RB, Lee NH (2010). Prostate apoptosis response protein 4 sensitizes human colon cancer cells to chemotherapeutic 5-FU through mediation of an NFκB and microRNA network. Molecular Cancer.

[28] Cerami E, Gao J, Dogrusoz U, Gross BE, Sumer SO, Aksoy BA, Jacobsen A, Byrne CJ, Heuer ML, Larsson E, Antipin Y, Reva B, Goldberg AP, Sander C, Schultz N (2012). The cBio Cancer Genomics Portal.

[29] Fabi F, Asselin E (2014). Expression, activation, and role of AKT isoforms in the uterus. Reproduction.

[30] Girouard J, Lafleur M-J, Parent S, Leblanc V, Asselin E (2013). Involvement of Akt isoforms in chemoresistance of endometrial carcinoma cells. Gynecologic Oncology.

[31] Gagnon V, Mathieu I, Sexton E, Leblanc K, Asselin E (2004). AKT involvement in cisplatin chemoresistance of human uterine cancer cells. Gynecol Oncol.

[32] Vara JÁF, Casado E, de Castro J, Cejas P, Belda-Iniesta C, González-Barón M (2004). PI3K/Akt signalling pathway and cancer. Cancer Treatment Reviews.

[33] Tan J, You Y, Xu T, Yu P, Wu D, Deng H, Zhang Y, Bie P (2014). Par-4 downregulation confers cisplatin resistance in pancreatic cancer cells via PI3K/Akt pathway-dependent EMT. Toxicology letters.

[34] Sharma AK, Kline CL, Berg A, Amin S, Irby RB (2011). The Akt Inhibitor ISC-4 Activates Prostate Apoptosis Response Protein-4 and Reduces Colon Tumor Growth in a Nude Mouse Model. Clinical Cancer Research.

[35] Chen X, Sahasrabuddhe AA, Szankasi P, Chung F, Basrur V, Rangnekar VM, Pagano M, Lim MS, Elenitoba-Johnson KSJ (2014). Fbxo45-mediated degradation of the tumor-suppressor Par-4 regulates cancer cell survival. Cell Death Differ.

[36] Thayyullathil F, Pallichankandy S, Rahman A, Kizhakkayil J, Chathoth S, Patel M, Galadari S (2013). Caspase-3 mediated release of SAC domain containing fragment from Par-4 is necessary for the sphingosine-induced apoptosis in Jurkat cells. Journal of Molecular Signaling.

[37] Treude F, Kappes F, Fahrenkamp D, Müller-Newen G, Dajas-Bailador F, Krämer OH, Lüscher B, Hartkamp J (2014). Caspase-8-mediated PAR-4 cleavage is required for TNFα-induced apoptosis. Oncotarget.

[38] Brasseur K, Auger P, Asselin E, Parent S, Côté J-C, Sirois M (2015). Parasporin-2 from a New Bacillus thuringiensis 4R2 Strain Induces Caspases Activation and Apoptosis in Human Cancer Cells. PLoS ONE.

[39] Guo N, Peng Z (2013). MG132, a proteasome inhibitor, induces apoptosis in tumor cells. Asia-Pacific Journal of Clinical Oncology.

[40] Drexler HCA (1997). Activation of the cell death program by inhibition of proteasome function. Proceedings of the National Academy of Sciences.

[41] Hornbeck PV, Zhang B, Murray B, Kornhauser JM, Latham V, Skrzypek E (2016). Par-4 phosphosite plus database. http://www.phosphosite.org/proteinAction.action?id=8332&showAllSites=true.

[42] Hornbeck PV, Zhang B, Murray B, Kornhauser JM, Latham V, Skrzypek E (2014). PhosphoSitePlus, 2014: mutations, PTMs and recalibrations. Nucleic acids research.

[43] Ao Li X, Ren J, Jin C, Xue Y (2009). BDM-PUB: computational prediction of protein ubiquitination sites with a Bayesian discriminant method.

[44] Radivojac P, Vacic V, Haynes C, Cocklin RR, Mohan A, Heyen JW, Goebl MG, Iakoucheva LM (2010). Identification, analysis, and prediction of protein ubiquitination sites. Proteins: Structure, Function, and Bioinformatics.

[45] Chen X, Qiu J-D, Shi S-P, Suo S-B, Huang S-Y, Liang R-P (2013). Incorporating key position and amino acid residue features to identify general and species-specific Ubiquitin conjugation sites. Bioinformatics.

[46] Davies BR, Greenwood H, Dudley P, Crafter C, Yu D-H, Zhang J, Li J, Gao B, Ji Q, Maynard J, Ricketts S-A, Cross D, Cosulich S, Chresta CC, Page K, Yates J (2012). Preclinical Pharmacology of AZD5363, an Inhibitor of AKT: Pharmacodynamics, Antitumor Activity, and Correlation of Monotherapy Activity with Genetic Background. Molecular Cancer Therapeutics.

[47] Hirai H, Sootome H, Nakatsuru Y, Miyama K, Taguchi S, Tsujioka K, Ueno Y, Hatch H, Majumder PK, Pan B-S (2010). MK-2206, an allosteric Akt inhibitor, enhances antitumor efficacy by standard chemotherapeutic agents or molecular targeted drugs *in vitro* and *in vivo*. Molecular cancer therapeutics.

[48] Kondapaka SB, Singh SS, Dasmahapatra GP, Sausville EA, Roy KK (2003). Perifosine, a novel alkylphospholipid, inhibits protein kinase B activation. Molecular Cancer Therapeutics.

[49] Prenzel T, Begus-Nahrmann Y, Kramer F, Hennion M, Hsu C, Gorsler T, Hintermair C, Eick D, Kremmer E, Simons M, Beissbarth T, Johnsen SA (2011). Estrogen-dependent gene transcription in human breast cancer cells relies upon proteasome-dependent monoubiquitination of histone H2B. Cancer Res.

[50] Wei Y, Jiang J, Liu D, Zhou J, Chen X, Zhang S, Zong H, Yun X, Gu J (2008). Cdc34-mediated Degradation of ATF5 Is Blocked by Cisplatin. Journal of Biological Chemistry.

[51] Gatti L, Hoe KL, Hayles J, Righetti SC, Carenini N, Bo LD, Kim DU, Park HO, Perego P (2011). Ubiquitin-proteasome genes as targets for modulation of cisplatin sensitivity in fission yeast. BMC Genomics.

[52] Fribley AM, Evenchik B, Zeng Q, Park BK, Guan JY, Zhang H, Hale TJ, Soengas MS, Kaufman RJ, Wang C-Y (2006). Proteasome Inhibitor PS-341 Induces Apoptosis in Cisplatin-resistant Squamous Cell Carcinoma Cells by Induction of Noxa. Journal of Biological Chemistry.

[53] Johnson CL, Lu D, Huang J, Basu A (2002). Regulation of p53 Stabilization by DNA Damage and Protein Kinase C. Molecular Cancer Therapeutics.

[54] Wang J, Zhou J-Y, Wu GS (2011). Bim Protein Degradation Contributes to Cisplatin Resistance. Journal of Biological Chemistry.

[55] Chanvorachote P, Nimmannit U, Stehlik C, Wang L, Jiang B-H, Ongpipatanakul B, Rojanasakul Y (2006). Nitric Oxide Regulates Cell Sensitivity to Cisplatin-Induced Apoptosis through S-Nitrosylation and Inhibition of Bcl-2 Ubiquitination. Cancer Research.

[56] Yang C, Kaushal V, Shah SV, Kaushal GP (2007). Mcl-1 is downregulated in cisplatin-induced apoptosis, and proteasome inhibitors restore Mcl-1 and promote survival in renal tubular epithelial cells. American Journal of Physiology - Renal Physiology.

[57] Johnstone RW, See RH, Sells SF, Wang J, Muthukkumar S, Englert C, Haber DA, Licht JD, Sugrue SP, Roberts T (1996). A novel repressor, par-4, modulates transcription and growth suppression functions of the Wilms' tumor suppressor WT1. Molecular and cellular biology.

[58] Cheema SK, Mishra SK, Rangnekar VM, Tari AM, Kumar R, Lopez-Berestein G (2003). Par-4 Transcriptionally Regulates Bcl-2 through a WT1-binding Site on the bcl-2 Promoter. Journal of Biological Chemistry.

[59] Nicholson KM, Anderson NG (2002). The protein kinase B/Akt signalling pathway in human malignancy. Cellular signalling.

[60] Risinger JI, Hayes K, Maxwell GL, Carney ME, Dodge RK, Barrett JC, Berchuck A (1998). PTEN mutation in endometrial cancers is associated with favorable clinical and pathologic characteristics. Clinical Cancer Research.

[61] Bruhn MA, Pearson RB, Hannan RD, Sheppard KE (2013). AKT-independent PI3-K signaling in cancer – emerging role for SGK3. Cancer Management and Research.

[62] Lee TJ, Lee JT, Kim SH, Choi YH, Song KS, Park JW, Kwon TK (2008). Overexpression of Par-4 enhances thapsigargin-induced apoptosis via down-regulation of XIAP and inactivation of Akt in human renal cancer cells. Journal of cellular biochemistry.

[63] Vivanco I, Sawyers CL (2002). The phosphatidylinositol 3-Kinase-AKT pathway in human cancer. Nat Rev Cancer.

[64] Pearce LR, Komander D, Alessi DR (2010). The nuts and bolts of AGC protein kinases. Nat Rev Mol Cell Biol.

[65] Lang F, Strutz-Seebohm N, Seebohm G, Lang UE (2010). Significance of SGK1 in the regulation of neuronal function. The Journal of Physiology.

[66] Das T, Suman S, Alatassi H, Ankem M, Damodaran C (2016). Inhibition of AKT promotes FOXO3a-dependent apoptosis in prostate cancer. Cell Death & Disease.

[67] Hodgkinson CP, Sale GJ (2002). Regulation of both PDK1 and the phosphorylation of PKC-ζ and-δ by a C-terminal PRK2 fragment. Biochemistry.

[68] Díaz-Meco MT, Municio MM, Frutos S, Sanchez P, Lozano J, Sanz L, Moscat J (1996). The Product of par-4, a Gene Induced during Apoptosis, Interacts Selectively with the Atypical Isoforms of Protein Kinase C. Cell.

[69] Joshi J, Fernandez-Marcos PJ, Galvez A, Amanchy R, Linares JF, Duran A, Pathrose P, Leitges M, Cañamero M, Collado M, Salas C, Serrano M, Moscat J, Diaz-Meco MT (2008). Par-4 inhibits Akt and suppresses Ras-induced lung tumorigenesis. The EMBO Journal.

[70] Lee TJ, Jang JH, Noh HJ, Park EJ, Choi KS, Kwon TK (2010). Overexpression of Par-4 sensitizes TRAIL-induced apoptosis via inactivation of NF-κB and Akt signaling pathways in renal cancer cells. Journal of cellular biochemistry.

[71] Asselin E, Mills GB, Tsang BK (2001). XIAP Regulates Akt Activity and Caspase-3-dependent Cleavage during Cisplatin-induced Apoptosis in Human Ovarian Epithelial Cancer Cells. Cancer Research.

[72] Basu A, Krishnamurthy S (2010). Cellular Responses to Cisplatin-Induced DNA Damage. Journal of Nucleic Acids.

[73] Nalca A, Qiu SG, El-Guendy N, Krishnan S, Rangnekar VM (1999). Oncogenic Ras Sensitizes Cells to Apoptosis by Par-4. Journal of Biological Chemistry.

[74] Barradas M, Monjas A, Diaz-Meco MT, Serrano M, Moscat J (1999). The downregulation of the pro-apoptotic protein Par-4 is critical for Ras-induced survival and tumor progression. EMBO J.

[75] Pereira MC, De Bessa-Garcia SA, Burikhanov R, Pavanelli AC, Antunes L, Rangnekar VM, Nagai MA (2013). Prostate apoptosis response-4 is involved in the apoptosis response to docetaxel in MCF-7 breast cancer cells. International Journal of Oncology.

[76] Ahn Y-T, Shin IJ, Kim J-M, Kim YS, Lee CHU, Ju S-A, An WG (2013). Counteracting the activation of pAkt by inhibition of MEK/Erk inhibition reduces actin disruption-mediated apoptosis in PTEN-null PC3M prostate cancer cell lines. Oncology Letters.

[77] Mendoza MC, Er EE, Blenis J (2011). The Ras-ERK and PI3K-mTOR pathways: cross-talk and compensation. Trends in Biochemical Sciences.

[78] Casolari DA, Pereira MC, de Bessa Garcia SA, Nagai MA (2011). Insulin-like growth factor-1 and 17beta-estradiol down-regulate prostate apoptosis response-4 expression in MCF-7 breast cancer cells. Int J Mol Med.

[79] Duong V, Boulle N, Daujat S, Chauvet J, Bonnet S, Neel H, Cavailles V (2007). Differential regulation of estrogen receptor alpha turnover and transactivation by Mdm2 and stress-inducing agents. Cancer Res.

[80] Lonard DM, Nawaz Z, Smith CL, O'Malley BW (2000). The 26S proteasome is required for estrogen receptor-alpha and coactivator turnover and for efficient estrogen receptor-alpha transactivation. Mol Cell.

[81] Sun M, Yang L, Feldman RI, Sun X-m, Bhalla KN, Jove R, Nicosia SV, Cheng JQ (2003). Activation of Phosphatidylinositol 3-Kinase/Akt Pathway by Androgen through Interaction of p85α, Androgen Receptor, and Src. Journal of Biological Chemistry.

[82] Guo R-X, Wei L-H, Tu Z, Sun P-M, Wang J-L, Zhao D, Li X-P, Tang J-M (2006). 17β-Estradiol activates PI3K/Akt signaling pathway by estrogen receptor (ER)-dependent and ER-independent mechanisms in endometrial cancer cells. The Journal of Steroid Biochemistry and Molecular Biology.

[83] Schneider CA, Rasband WS, Eliceiri KW (2012). NIH Image to ImageJ: 25 years of image analysis. Nature methods.

