# Supplementary material for: Post-translational regulation of the cleaved fragment of Par-4 in ovarian and endometrial cancer cells
Source: Oncotarget. 2016 May 9;7(24):36971–87. doi: 10.18632/oncotarget.9235 (PMC5095052; doi:10.18632/oncotarget.9235)
Supplement: Supplementary file 1 [file oncotarget-07-36971-s001.pdf]

Post-translational regulation of the cleaved fragment of Par-4 in ovarian and endometrial cancer cells

SUPPLEMENTARY FIGURE

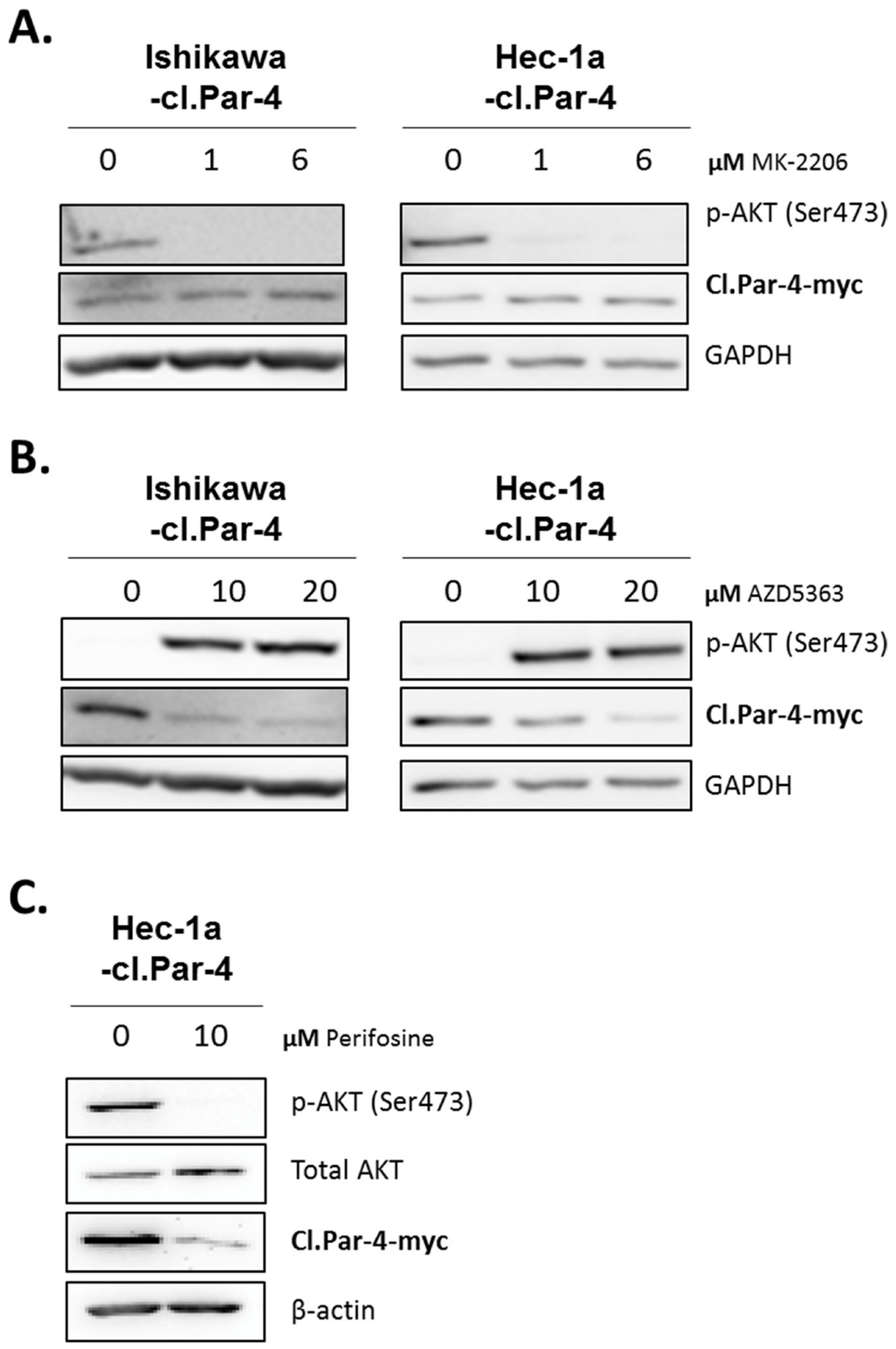

Supplementary Figure S1: AKT inhibition does not increase cleaved-Par-4 protein level. Cl-Par-4 cancer cells were treated with either **A.** 1 or 6µM AKT inhibitor (MK-2206), **B.** 10 or 20µM AKT inhibitor (AZD-5363) or **C.** 10µM of AKT inhibitor (Perifosine) for 24h. The levels of p-AKT (Ser473), total AKT and cl-Par-4-myc were determined in treated cells using western blot analysis. GAPDH or β-actin were used as loading controls. Results shown are representative of three independent experiments.
